# Complex Regional Pain Syndrome: Diagnosis, Pathophysiology, and Treatment Approaches

**DOI:** 10.7759/cureus.76324

**Published:** 2024-12-24

**Authors:** Bruno Lima Pessôa, José Geraldo M Netto, Lorena Adolphsson, Lucas Longo, Wilhelmina N Hauwanga, Billy McBenedict

**Affiliations:** 1 Neurosurgery, Fluminense Federal University, Niterói, BRA; 2 Cardiology, Gaffrée and Guinle University Hospital, Federal University of the State of Rio de Janeiro, Rio de Janeiro, BRA

**Keywords:** central sensitization, complex regional pain syndrome (crps), neuropathic pain, peripheral sensitization, spinal cord stimulation

## Abstract

Complex regional pain syndrome (CRPS) is a chronic pain condition characterized by significant sensory, motor, and autonomic dysfunction, often following trauma or nerve injury. Historically known as causalgia and reflex sympathetic dystrophy, CRPS manifests as severe, disproportionate pain, often accompanied by hyperalgesia, allodynia, trophic changes, and motor impairments. Classified into type I (without nerve injury) and type II (associated with nerve damage), CRPS exhibits a complex pathophysiology involving peripheral and central sensitization, neurogenic inflammation, maladaptive brain plasticity, and potential autoimmune and psychological influences. The diagnosis relies primarily on clinical evaluation using criteria such as the Budapest Criteria, supported by supplementary tests to exclude differential diagnoses. However, its overlapping features with other conditions complicate diagnostic accuracy.

The management of CRPS necessitates a multidisciplinary approach combining physical therapy, psychological support, and pharmacotherapy. Physical therapies, including graded motor imagery and mirror therapy, are essential for preserving function and preventing complications. Pharmacological treatments target neuropathic pain and inflammatory components, utilizing agents such as gabapentinoids, corticosteroids, and bisphosphonates. In refractory cases, interventional modalities like spinal cord stimulation and dorsal root ganglia stimulation provide promising options, although their efficacy remains variable. Emerging therapies, such as immune-modulatory treatments and advanced neuromodulation techniques, reflect the ongoing pursuit of effective interventions. This review synthesizes current knowledge, providing insights into diagnostic frameworks, pathophysiological mechanisms, and evolving treatment strategies to improve outcomes for individuals affected by CRPS.

## Introduction and background

In 1864, Mitchell, a physician during the American Civil War, described a condition affecting 10% of patients with partial peripheral nerve damage in the distal limbs. This condition, characterized by severe, difficult-to-treat distal burning pain, was termed "causalgia." Mitchell emphasized the partial nature of the nerve injury required for the syndrome to occur, describing it as involving sensory and trophic abnormalities that extended beyond the innervation territory of the injured nerve, sometimes appearing far from the injury site [1]. During the interwar period (World Wars I and II), the theory that pain could be sustained by the sympathetic nervous system (SNS) in patients without diagnosed nerve damage gained traction. Patients developed extremity pain accompanied by edema. This phenomenon was first described by Paul Sudeck, who identified precipitating factors, namely fractures, mild trauma, low-grade infections, burns, acute myocardial infarction, or stroke. This syndrome became known as reflex sympathetic dystrophy [2].

Complex regional pain syndrome (CRPS) is a chronic, painful neuropathy characterized by significant autonomic alterations that arise following acute tissue trauma. The term "CRPS" has replaced previously used terminologies, including reflex sympathetic dystrophy, causalgia, and others [3]. Clinically, CRPS is marked by typical neuropathic pain symptoms, including burning pain, hyperalgesia, and allodynia. These symptoms are often accompanied by autonomic disturbances, such as changes in skin temperature, sweating, and discoloration at the site of pain [4]. Trophic changes, such as alterations in hair growth, nail appearance, and skin texture, are also common. Motor changes, including paresis, reduced range of motion, and tremors, are frequently observed [5]. Approximately half of CRPS patients exhibit intention or postural tremor, which reflects an increase in physiological tremor. Additionally, some patients may develop dystonia in the affected hand [6].

From an etiological perspective, CRPS is divided into two types: type I and type II. Type I corresponds to the former term "reflex sympathetic dystrophy" and is characterized by the absence of a documented anatomical lesion. In contrast, CRPS type II, previously referred to as causalgia, is associated with documented nerve damage [5]. CRPS type I can result from various injuries, ranging from minor tissue trauma to fractures, sprains, strains, surgical procedures, or even spinal cord and brain injuries [4]. Although the classification into two subtypes serves a didactic purpose, clinically, the distinction is minimal except for the presence of a peripheral nerve injury in CRPS type II. This injury is accompanied by a focal nerve deficit, which is a mandatory criterion for diagnosis. Despite this distinction, the pathophysiology and treatment of both subtypes are fundamentally the same [7].

In the United States, it is estimated that 50,000 new cases are diagnosed annually. A European study reported a slightly higher incidence, with rates up to 26.2%. CRPS is more prevalent in women, with a female-to-male ratio ranging from 2:1 to 4:1. It affects individuals of all ages, with an average age of 40-50 years at diagnosis, and peaks in incidence between 60 and 70 years. Regarding subtypes, CRPS I is more common than CRPS II, with the latter accounting for 2-14% of cases of peripheral nerve injury. Retrospective studies suggest an upper limb-to-lower limb ratio of 1:1 to 2:1 [8,9]. Beyond the painful condition itself, CRPS often leads to significant functional and psychological impairments [10]. From a treatment standpoint, it is one of the most challenging and refractory pain syndromes to manage [11]. Currently, no randomized studies demonstrate the effectiveness of interventional treatments for this condition [12].

However, given the limitations of clinical treatments, various palliative and interventional approaches have been developed. These include drug infusion pumps and spinal cord stimulation (SCS), both of which are increasingly employed. Electrophysiological studies (e.g., electromyography and nerve conduction) are generally normal, except in chronic stages of the disease. Similarly, imaging studies are usually unremarkable [6]. Thus, CRPS remains a poorly understood and challenging condition, characterized by diverse clinical manifestations and an unclear pathophysiological basis. The variability in symptoms and overlapping features with other conditions complicate diagnosis, while treatment options often yield inconsistent outcomes. These challenges highlight the critical need for a clear understanding of CRPS's underlying mechanisms and the development of effective, evidence-based management strategies. This review aimed to synthesize current knowledge on CRPS, offering insights into its diagnostic framework, emerging theories of pathophysiology, and evolving therapeutic approaches. By discussing recent advancements, this review sought to provide a foundation for improved diagnostic accuracy and therapeutic approaches.

## Review

The discussion critically examines the diagnostic complexities and treatment limitations of CRPS, emphasizing its diverse clinical manifestations and poorly understood pathophysiology. It highlights the utility and limitations of the Budapest Criteria in distinguishing CRPS from other neuropathic conditions and describes the available interventions, including pharmacological agents, physical therapies, and advanced neuromodulation techniques.

Complexity of CRPS

Pathophysiology

The pathogenesis of CRPS remains unclear, with proposed mechanisms involving both peripheral and central nervous systems, including classic inflammation, neurogenic inflammation, and maladaptive changes in pain perception and somatosensory processing [13]. Despite numerous efforts to define its pathophysiology, no single theory fully explains the condition. It is widely accepted that CRPS arises from multiple mechanisms, which account for its varied presentations [14,15]. This multifactorial nature is supported by recent findings showing that CRPS is not solely a sympathetically mediated disease but also involves central nervous system alterations. Additionally, somatomotor changes and bilateral tactile, thermal, and pain abnormalities have been observed in patients with unilateral CRPS [14]. Among the proposed pathophysiological mechanisms are alterations in cutaneous innervation following injury, central and peripheral sensitization, changes in the SNS, and the involvement of circulating catecholamines. Other contributing factors include a proinflammatory state, brain plasticity, genetic predispositions, and psychological influences. These factors are thought to interact synergistically, forming the complex pathophysiological foundation of CRPS [7].

Changes in Cutaneous Innervation After Injury

This mechanism has been observed even in patients with CRPS I, where no nerve injury is described. It is postulated that an initial trauma triggers a cascade of events leading to CRPS. Supporting evidence comes from skin biopsy studies, which have demonstrated a reduction in the density of C and A delta fibers in affected limbs compared to pain-free areas on the same extremity and in healthy individuals [16].

Alteration of the Sympathetic Nervous System

Studies in guinea pigs have demonstrated that following an initial nerve injury, there is an increase in the expression of adrenergic receptors in nociceptive fibers, which may explain why heightened sympathetic activity can trigger nociceptive stimuli [17,18]. However, the only prospective study in humans examining the correlation between CRPS and increased SNS activity failed to confirm this theory. Schurman et al. proposed that during the acute phase of CRPS, there is an early decrease in sympathetic activity, which, when present, predicts the onset of CRPS following trauma [18]. Furthermore, the study found a decrease in sympathetic activity in unaffected limbs, suggesting that the sympathetic dysfunction observed in CRPS is diffuse and not confined to the affected limb.

Peripheral Sensitization

Following initial trauma, primary afferent fibers in the injured area release nociceptive substances like substance P and bradykinin. These inflammatory mediators increase the baseline firing frequency of nociceptors and lower their response thresholds to thermal and mechanical stimuli, contributing to hyperalgesia and allodynia in affected patients [17,19].

Brain Plasticity

Recent research on functional imaging and pain has provided insights into the neural mechanisms underlying CRPS. Several studies have shown evidence of reorganization in somatotopic areas of the brain in CRPS patients. Specifically, a reduction in the size of the somesthetic area representation has been observed in these patients compared to healthy individuals [20,21]. Two studies have further demonstrated that, following successful treatment of CRPS, these changes in the somesthetic area normalize, suggesting that these alterations reflect brain plasticity resulting from the condition [22,23]. Additionally, the degree of cortical reorganization has been found to correlate with the severity of pain and hyperalgesia [20].

Role of Circulating Catecholamines

Excessive vasoconstriction caused by cold in the affected limb may lead to further vasoconstriction, raising the hypothesis of an excess of circulating catecholamines [24]. However, current evidence shows that catecholamine levels are actually decreased, and the vasoconstriction is instead due to increased expression of catecholamine receptors in nociceptive fibers compared to the unaffected side [25]. This phenomenon is speculated to result from decreased SNS activity, which induces an "upregulation" of catecholamine receptors [25-28].

Inflammatory Substances

The clinical improvement observed in some cases with corticosteroid treatment suggests that inflammatory peptides play a significant role in the development of CRPS [29]. This process likely originates from a classic inflammatory mechanism involving mast cells and lymphocytes activated after a triggering event, such as trauma. These cells produce cytokines (IL-1β and IL-6) and tumor necrosis factors (TNF-α), which contribute to the edema commonly observed in CRPS [19,30]. Furthermore, nociceptive fibers, in response to nerve injury, can release cytokines and inflammatory peptides, such as bradykinin, substance P, and calcitonin, leading to what is known as neurogenic inflammation [31,32].

Autoimmune Regulation

A significant number of CRPS patients have autoantibodies against autonomic neuron surface epitopes, suggesting autoimmunity plays a role in CRPS pathogenesis [33]. These autoantibodies, primarily IgG and IgM, target nervous system components like sympathetic neurons and adrenergic receptors, as shown in both patient and animal models [30,34]. Animal studies also demonstrated that passive transfer of CRPS patient IgG enhances pain sensitivity and motor dysfunction [35].

Goebel and Blaes proposed that limb injury creates conditions that allow circulating IgG autoantibodies to bind to previously hidden or altered target structures by disrupting the blood-nerve barrier and revealing new epitopes [36]. Thus, findings suggest that CRPS may be an autoimmune syndrome, with the potential for targeted therapies to modulate the immune response and improve patient outcomes.

Genetic Factors

Although some studies suggest a genetic component in the development of CRPS, including reports of familial cases, these findings are based on small patient samples, limiting their reliability in establishing a causal relationship [37,38]. Research has identified polymorphisms in genes encoding α1a adrenoceptors and the HLA system [39,40]. A study identified genetic differences in CRPS, with HLA-B62 linked to CRPS with dystonia and HLA-DQ8 associated with both forms [41]. Further research is needed to confirm the role of genetics in this condition.

Psychological Factors

Based on the theory that adrenergic discharges can exacerbate the clinical condition of CRPS, and considering that psychological stress is associated with the release of catecholamines into the bloodstream, it can be inferred that psychological factors play a significant role in the condition [42,43]. Additionally, recent studies have highlighted the relationship between psychological factors and a patient's immunological status [44,45]. This suggests that psychological conditions may directly influence the pro-inflammatory state associated with CRPS.

Diagnosis

Clinical Evaluation

The diagnosis of CRPS (ICD-11: MG30.04) is primarily clinical, suspected in the presence of persistent pain that is disproportionate to the expected recovery from trauma or injury [46,47]. The pain is regional, not confined to a specific nerve or dermatome, and is often accompanied by symptoms such as hyperalgesia, allodynia, temperature asymmetry, changes in skin color, edema, altered sweating, motor dysfunction, and trophic changes in the affected limb [47]. However, the lack of universally accepted diagnostic criteria often leads to confusion with other painful syndromes of the extremities.

For clinical evaluation, a diagnostic test with excellent sensitivity and good specificity is essential, while for research purposes, specificity is paramount. A consensus-based criteria, known as Budapest Criteria, was established by the International Association for the Study of Pain (IASP) as a revision of earlier versions [3]. Accordingly, patients must report continuous pain disproportionate to any inciting event and present at least one symptom in three of the following four categories: sensory (e.g., hyperesthesia, allodynia), vasomotor (e.g., temperature asymmetry, skin color changes), sudomotor/edema (e.g., sweating changes, edema), and motor/trophic (e.g., decreased range of motion, dystonia, or trophic changes) (Table 1). Patients must also exhibit at least one sign in two of these categories during evaluation, and other conditions that could better explain the symptoms must be excluded. Compared to the previous ISAP diagnostic criteria [48], the Budapest criteria demonstrated higher sensitivity (0.99) and specificity (0.68) in distinguishing CRPS from other neuropathic pain conditions [13].

**Table 1:** Clinical diagnostic criteria for CRPS The table is based on the criteria revised by the IASP in 2012 [3]. CRPS: complex regional pain syndrome, IASP: International Association for the Study of Pain

Criterion	Description
1	Continuing pain, which is disproportionate to any inciting event
2	Must report at least one symptom in three of the four following categories: (1) sensory: reports of hyperalgesia and/or allodynia; (2) vasomotor: reports of temperature asymmetry and/or skin color changes and/or skin color asymmetry; (3) sudomotor/edema: reports of edema and/or sweating changes and/or sweating asymmetry; (4) motor/trophic: reports of decreased range of motion and/or motor dysfunction (weakness, tremor, dystonia) and/or trophic changes (hair, nail, skin)
3	Must display at least one sign at the time of evaluation in two or more of the following categories: (1) sensory: evidence of hyperalgesia (to pinprick) and/or allodynia (to light touch and/or deep somatic pressure and/or joint movement); (2) vasomotor: evidence of temperature asymmetry and/or skin color changes and/or asymmetry; (3) sudomotor/edema: evidence of edema and/or sweating changes and/or sweating asymmetry; (4) motor/trophic: evidence of decreased range of motion and/or motor dysfunction (weakness, tremor, dystonia) and/or trophic changes (hair, nail, skin)
4	There is no other diagnosis that better explains the signs and symptoms

Supplementary Tests

Supplementary tests are often employed to support the diagnosis of CRPS in atypical cases or to rule out alternative explanations for symptoms. Three-phase bone scintigraphy can identify increased radiotracer uptake in joints distant from the trauma site, particularly when performed within the first five months of symptom onset [49,50]. Plain radiographs may reveal spotty bone decalcification, although this finding lacks high sensitivity. Autonomic testing, as seen in assessments of resting skin temperature or sweat output, can provide additional evidence [51], but their practicality is limited due to the complexity and duration of testing [52].

Magnetic resonance imaging (MRI) and computed tomography (CT) scans are not routinely recommended as they do not confirm the diagnosis [53]. While MRI can assist in ruling out other conditions, CT scans have limited utility, involve higher costs compared to plain radiographs, and expose patients to radiation. Although these tests can aid in the diagnostic process, the diagnosis of CRPS remains primarily clinical, as no neurophysiological test has demonstrated both sufficient sensitivity and specificity.

Historically, pain relief following sympathetic nerve blocks was considered diagnostic. However, this approach is no longer widely accepted due to inconsistent evidence regarding the role of the SNS in CRPS. Modern understanding interprets such responses as indicative of sympathetically maintained pain rather than definitive proof of CRPS. Changes in nerve conduction studies and somatosensory evoked potentials may reflect nerve dysfunction, but neuropathic symptoms such as dysesthesias are primarily attributed to small fiber damage, which also plays a key role in the painful condition [54,55].

Differential Diagnoses

An accurate diagnosis of CRPS requires carefully considering and excluding various differential diagnoses [56-58]. Spontaneous pain onset, fever, and abnormal lab results may suggest infection or inflammatory diseases like rheumatoid arthritis. Neurological abnormalities, including signs of central or peripheral nerve lesions, could point to conditions like spinal cord tumors, stroke, or nerve compression. A history of malignancy, B symptoms, or involvement of multiple limbs raises suspicion for paraneoplastic disorders. Pain that spreads occurs during weight-bearing or responds unusually well to simple analgesics may indicate osteoarthritis, myofascial pain, or prior bony or soft tissue injury. Vascular issues, including venous thrombosis, vasculitis, or Raynaud’s disease, should be considered, particularly when signs of acute vascular changes, such as arterial insufficiency or lymphatic obstruction, are present. Additionally, conditions like compartment syndrome, thoracic outlet syndrome, or erythromelalgia should be considered. Psychological factors, including factitious disorder or malingering related to compensation claims, especially in cases of self-harm or Gardner-Diamond syndrome, must also be evaluated. Early-phase differential diagnoses should focus on infections, neurological compression, and inflammatory conditions, as these can closely mimic the symptoms of CRPS and require careful differentiation.

Treatment

Multidisciplinary Approach

The management of CRPS involves several core interventions aimed at addressing pain, preserving function, and promoting psychological well-being. Physical and occupational therapy, including mirror therapy and graded motor imagery, considered first-line treatments [47,59], should be initiated promptly to preserve the range of motion and prevent complications like contractures. Common techniques include general exercises, transcutaneous electrical nerve stimulation, gait retraining, hydrotherapy, and relaxation strategies [47,60]. Psychological therapy is crucial, particularly for patients with prolonged symptoms, poor response to initial therapies, or suspected psychological comorbidities. Referrals to clinical psychologists with CRPS expertise are recommended for addressing anxiety, depression, counterproductive behaviors, or family dynamics that may perpetuate disability [61,62]. Although cognitive behavioral therapy is not extensively studied in CRPS, its efficacy in other chronic pain conditions supports its use.

Pharmacotherapy

Polypharmacy is often necessary to address the diverse symptoms and subtypes of CRPS, although monotherapy is preferred to minimize adverse effects, cost, and patient non-compliance [17]. Ideally, medications that target multiple symptoms, such as tricyclic antidepressants, which relieve neuralgic pain and help manage anxiety, depression, and insomnia, are used [39,63]. Nonsteroidal anti-inflammatory drugs were previously used to target pain and inflammation in CRPS, but recent evidence shows limited benefit, with no statistically significant improvement in symptoms [64]. Adjunctive neuropathic pain medications may include gabapentin, pregabalin, antidepressants (tricyclics and serotonin reuptake inhibitors), sodium channel blockers (e.g., carbamazepine, lamotrigine), opioids, steroids, immunomodulatory therapies (e.g., infliximab), immunoglobulins, and calcium-regulating drugs. Except for steroids and gabapentin, the evidence supporting the efficacy of many of these treatments in CRPS is limited [65-67]. Bisphosphonates are an option for patients with abnormal bone scans, supported by several small randomized trials demonstrating pain reduction [68-70]. Topical treatments with lidocaine can be used for mild to moderate pain, though their efficacy is also limited [71].

Opioids are considered only in refractory cases with careful risk-benefit assessment, which has been shown in clinical practice to be of great help when other agents are ineffective [65-67]. For patients unresponsive to initial therapies, higher-risk treatments, including calcitonin, glucocorticoids, and ketamine infusions, may be considered. Calcitonin, despite weak evidence, has been shown to reduce bone resorption and may offer analgesic effects in combination with physical therapy [72]. Glucocorticoids are associated with significant improvement in outcomes, although some studies reported minor side effects, and only one described major adverse events [73]. Schwartzman et al. demonstrated that intravenous ketamine administered in an outpatient setting led to significant reductions in various pain parameters [74]. Similarly, a multiple-day ketamine infusion provided significant pain relief in chronic CRPS-1 patients but did not improve function [75]. According to a study, prolonged dosing with ketamine may increase the likelihood of therapeutic benefit. Moreover, ketamine treatment was generally safe, with psychomimetic side effects that were well-tolerated by most patients [76].

Interventional Options

Interventional treatments for refractory pain are reserved for patients who have not experienced significant improvement with other types of treatments [47]. These interventions are typically introduced progressively, becoming more invasive if the initial therapies do not result in improvement within approximately two weeks. Common interventional therapies include tender point injections, nerve blocks, SCS, epidural clonidine, and sympathectomies. Despite the limited evidence, many clinicians report meaningful benefits for certain patients from these interventions, highlighting the importance of individualized treatment approaches. Although their efficacy is controversial, sympathetic blocks have been widely used, primarily in specialized centers [77,78]. This conflicting evidence underscores the need for careful patient selection and further research to determine the efficacy of sympathetic nerve blocks in CRPS management. An alternative sympathetic block method, the Bier block, uses antiadrenergic drugs administered into a vein near the sympathetic ganglia with a tourniquet applied to occlude circulation. Limited evidence is available for the use of surgical sympathectomy, either surgically or by thoracoscopy [74,79].

Implanted devices, such as SCS and peripheral nerve stimulation (PNS), offer neuromodulation strategies that may benefit patients with CRPS when traditional therapies fail [80,81]. These approaches are particularly considered for patients with disease confined to one extremity. SCS reduces pain and improves health-related quality of life, although function is not improved [10,80,82,83]. PNS has also been reported as a safe and efficacious treatment, with improved function and reduced long-term pain [84-86]. In a smaller study, direct electrical stimulation of the sciatic nerve effectively controlled disabling foot pain, dystonia, and autonomic features in CRPS type I patients who had failed SCS [87]. Dorsal root ganglia (DRG) stimulation, an emerging alternative to SCS and PNS, is increasingly used as a first choice when these techniques fail. Data support that DRG stimulation may offer superior outcomes compared to SCS, though the risks and complications are similar [88,89]. These complications, including improper electrode positioning, highlight the importance of performing these procedures in expert centers [80,90].

Limitations

This review provides a comprehensive synthesis of current diagnostic, pathophysiological, and treatment approaches. However, it has some limitations. The variability in CRPS presentations and the lack of universally accepted diagnostic criteria introduce a degree of subjectivity that complicates comparing studies. Furthermore, the reliance on clinical diagnostic tools, such as the Budapest Criteria, may overlook atypical cases or misclassify other conditions. While emerging therapies are highlighted, their efficacy remains preliminary and requires further validation through randomized controlled trials. Finally, the review does not address long-term outcomes or the cost-effectiveness of advanced interventions, which are critical for broader clinical applications.

## Conclusions

CRPS represents a multifaceted and challenging condition marked by persistent pain and significant autonomic, motor, and trophic changes. Despite advancements in understanding its diagnostic criteria, pathophysiology, and treatment, CRPS remains a clinical enigma with no universally effective cure. The interplay of peripheral and central mechanisms, neurogenic and classic inflammation, and potential autoimmune and psychological influences underscores its complex etiology. Diagnosis primarily relies on clinical evaluation supported by consensus-based criteria, such as the Budapest Criteria, while supplementary tests aid in atypical presentations or differential diagnoses. Current treatment approaches emphasize a multidisciplinary strategy combining physical therapy, psychological interventions, pharmacotherapy, and interventional modalities tailored to individual patient needs. Emerging techniques, such as DRG stimulation, show promise but require further advancements to establish efficacy and safety. Future research should focus on CRPS’s underlying mechanisms, optimizing diagnostic tools, and developing targeted therapies to improve patient outcomes.
